# Calcitriol induces cell senescence of kidney cancer through JMJD3 mediated histone demethylation

**DOI:** 10.18632/oncotarget.22124

**Published:** 2017-10-26

**Authors:** Yongqing Shen, Dan Yu, Pan Qi, Xuliang Wang, Xiaoqiang Guo, Aili Zhang

**Affiliations:** ^1^ Department of Urology, The Fourth Hospital of Hebei Medical University, Shijiazhuang 050035, Hebei, China; ^2^ Department of Nursing, Hebei University of Chinese Medicine, Shijiazhuang 050020, Hebei, China; ^3^ State Engineering Laboratory of Medical Key Technologies Application of Synthetic Biology, Key Laboratory of Medical Reprogramming Technology, Shenzhen Second People’s Hospital, The First Affiliated Hospital of Shenzhen University, Shenzhen 518035, Guangdong, China; ^4^ Department of Urology, Peking University Shenzhen Hospital, Institute of Urology of Shenzhen PKU-HKUST Medical Center, Shenzhen 518036, Guangdong, China; ^5^ Kidney Disease Center, The First Affiliated Hospital, College of Medicine, Zhejiang University, Hangzhou 310003, Zhejiang, China; ^6^ Longgang District Central Hospital, Shenzhen 518116, Guangdong, China

**Keywords:** kidney cancer, calcitriol, cell senescence, JMJD3, p16INK4A

## Abstract

Calcitriol, also known as 1,25-dihydroxyvitamin D3 (1,25(OH)_2_VD_3_), is a biologically active form of vitamin D and has a wide range of anticancer activity against various cancer cell lines. However, the mechanism of calcitriol remains to be further studied. In this study, the biological effect and epigenetic regulation of calcitriol on kidney cancer cells were investigated. Calcitriol can significantly inhibit cell proliferation of kidney cancer cell lines 786-O (*P*<0.05). Calcitriol also induced cell apoptosis and senescence of 786-O and ACHN (*P*<0.05). Calcitriol can increase the expression of histone demethylase JMJD3 and cell senescence marker p16INK4A (*P*<0.05). Knockdown of JMJD3 decreased p16INK4A upregulation after calcitriol treatment (*P*<0.05), and also reduced calcitriol-induced cell senescence (*P*<0.05). This study reveals a new mechanism of anticancer activity of calcitriol by showing that histone demethylase JMJD3 induced by calcitriol increases p16INK4A expression and cell senescence. Therefore, these results provide new strategy for treatment and prevention of kidney cancer.

## INTRODUCTION

Kidney cancer is one of the most lethal type of genitourinary cancer, accounts for ∼3% of human malignancies [1]. The global incidence of kidney cancer has increased over the past two decades by 2% per year [2]. Kidney cancer remains hard to be detected, difficult to be treated and poorly understood [3], so there is slow in early diagnosis and treatment of kidney cancer.

Kidney cancer is generally resistant to chemotherapy and radiation therapy [4]. The effects of target therapy for kidney cancer, including sorafenib, sunitinib, bevacizuma and temsirolimus, are also very limited [5]. Therefore, it is required new therapeutic targets or treatment modalities.

Vitamin D is not only a fat-soluble vitamin which is important for calcium absorption and bone development, but also an important hormone which regulates cellular differentiation and proliferation in normal and malignant cells [6]. Several studies have determined that low blood levels of vitamin D3 are correlated with increased risk of development of certain cancers including digestive-system cancers and colon cancer [7, 8], so vitamin D3 supplementation is beneficial to cancer prevention [9, 10]. Preclinical and clinical studies suggest lower vitamin D levels are associated with worse outcomes and higher vitamin D levels associated with better outcomes [11, 12]. The results indicated that vitamin D supplement is also an economical and safe way to improve cancer prognosis and outcome [13].

Some researches indicated that vitamin D also plays important effect on urological cancers development [14]. Higher vitamin D levels are associated with a lower risk of kidney cancer in men and women [15]. Kidney is a primary production organ for vitamin D biologically active form, calcitriol, also known as 1,25-dihydroxyvitamin D_3_ (1,25(OH)_2_VD_3_) [10], so the relationship between vitamin D and kidney cancer may be more closely related. Therefore, further investigation on the mechanism of vitamin D3 will be of great value for prevention and prognosis of kidney cancer. Recent studies showed that calcitriol regulates the expression of histone demethylase JMJD3 [16, 17], which expands the understanding on the role of calcitriol.

In this study, we investigated the role and mechanism of calcitriol on kidney cancer cell lines. The results indicated that calcitriol can inhibit cell proliferation and induce cell apoptosis and senescence. Our results also demonstrated that calcitriol can up-regulate the expressions of histone H3K27 demethylase JMJD3 and cell senescence marker p16INK4A. Inhibition of JMJD3 can decrease calcitriol-induced cell senescence and p16INK4A up-regulation. All these indicted that JMJD3 mediated cell senescence may play an important role in the anticancer activity of calcitriol.

## RESULTS

### The expressions of JMJD3 and p16INK4A are positively correlated in kidney cancer

Our previous experiments have demonstrated that the expression of JMJD3 was increased in cancer tissues of kidney cancer compared to adjacent tissues [18]. In this study, the mRNA levels of JMJD3 and cell senescence marker p16INK4A were further determined using qPCR. The results indicated that both *JMJD3* and *p16INK4A* were overexpressed in cancer tissues compared to adjacent tissues (*P* < 0.05, Supplementary Figure 1A). There was a positive correlation between the mRNA levels of *JMJD3* and *p16INK4A* (Supplementary Figure 1B). The results also indicated that the level of H3K27me3 was decreased significantly in cancer tissues with the increase of JMJD3 protein (*P* < 0.05, Supplementary Figure 1C), and there was a negative correlation between them (Supplementary Figure 1D). All these data mean that both JMJD3 catalyzed histone demethylation and cell senescence may be important in kidney cancer development.

### Calcitriol inhibits cell proliferation of kidney cancer

**Figure 1:**
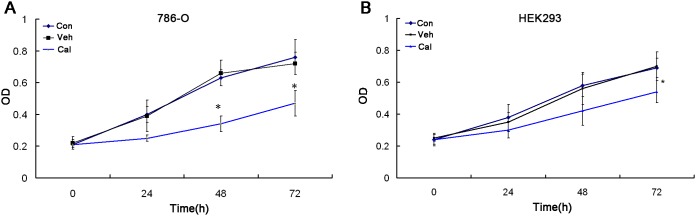
Calcitriol inhibits cell proliferation of kidney cancer cells 786-O **(A)** and HEK293 **(B)** cells were treated with vehicle (Veh) or 100nM calcitriol (Cal) for 24h, 48h and 72h, and untreated group as control (Con). The effects on cell proliferation were determined with MTT assay. *, *P* < 0.05.

MTT assay indicated that calcitriol can significantly inhibit cell proliferation of 786-O cells at 100nM (*P*<0.05, Figure 1A). However, the inhibitory effect of calcitriol on HEK293 was moderate (*P*<0.05, Figure 1B). Previous researches showed that calcitriol can decrease cell proliferation of kidney cancer [19], and our results further confirm this conclusion.

### Calcitriol promotes cell apoptosis of kidney cancer

**Figure 2:**
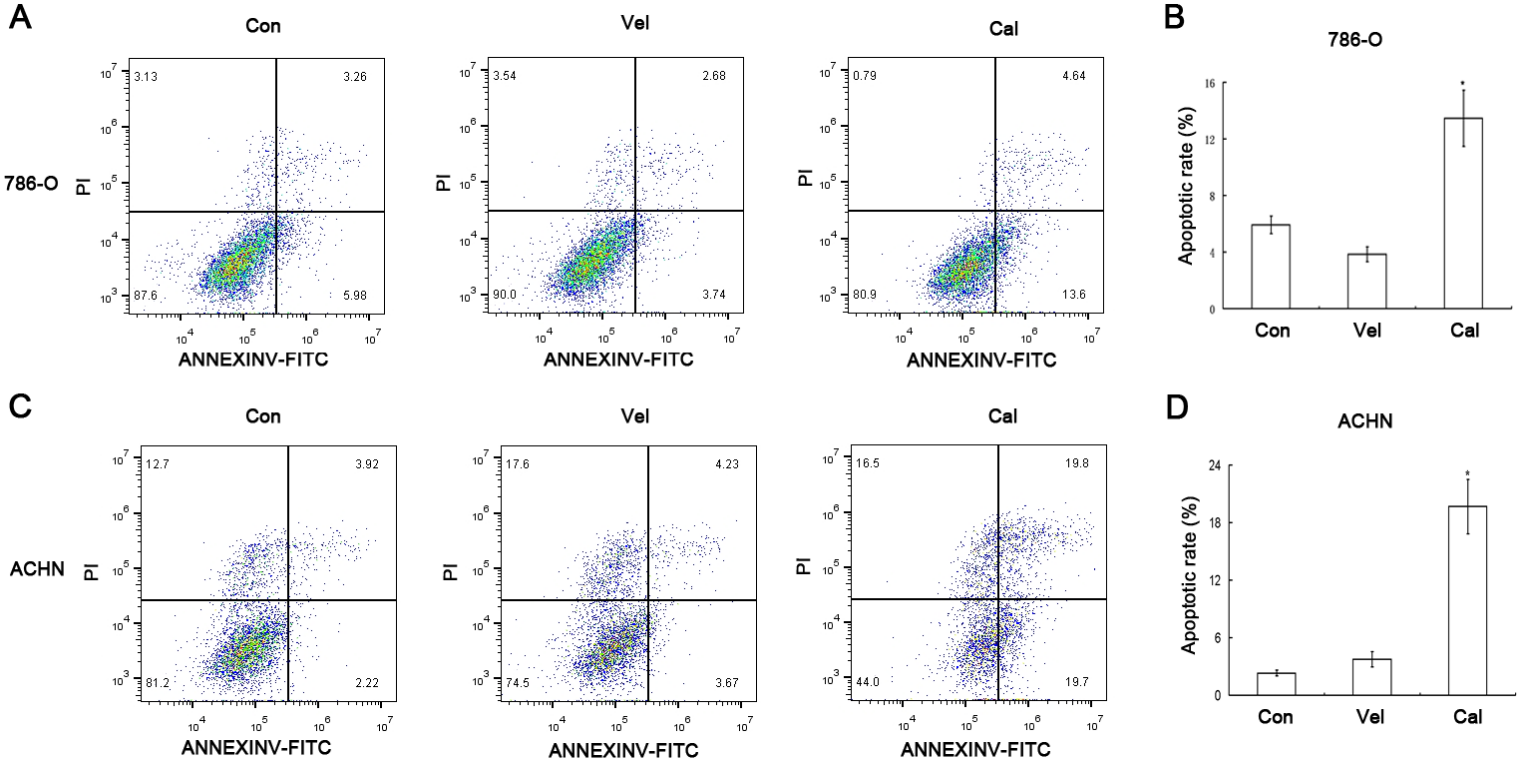
Calcitriol promotes the apoptosis of kidney cancer cells 786-O and ACHN cells were treated with vehicle (Veh) or 100nM calcitriol (Cal) for 48h, and untreated group as control (Con). The cell apoptosis rates were determined by flow cytometry analysis. (**A)** 786-O. (**B)** Quantitative results of 786-O cell apoptosis. (**C)** ACHN. (**D)** Quantitative results of ACHN cell apoptosis. Scale bar, 100μm. Magnification, 200×. *, *P* < 0.05.

The flow cytometry analysis indicated that calcitriol induced cell apoptosis of 786-O and ACHN cells (Figure 2A and 2C), and there were significant differences (*P*<0.05, Figure 2B and 2D). Previous studies have shown that calcitriol can stimulate cell apoptosis of multiple types of cancer cells including breast and prostate cancer cell lines [20, 21]. In the study, our results further suggest that calcitriol is also effective against kidney cancer cells.

### Calcitriol induces cell senescence of kidney cancer

**Figure 3:**
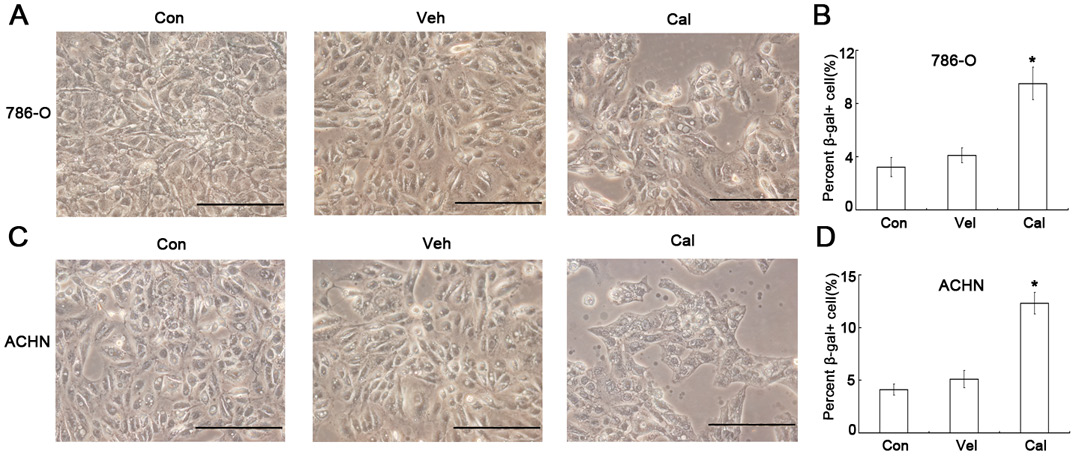
Calcitriol induces cell senescence of kidney cancer cells 786-O and ACHN cells were treated with vehicle (Veh) or 100nM calcitriol (Cal) for 48h, and untreated group as control (Con). The cell senescence was determined by SA-β-gal staining. (**A)** 786-O. (**B)** Quantitative results of 786-O cell senescence. (**C)** ACHN. **(D)** Quantitative results of cell senescence. *, *P* < 0.05.

To understand other biological effects of calcitriol, we measured cell senescence of kidney cancer cells 786-O and ACHN after calcitriol treatment. The data indicated that calcitriol can significantly increase the percentage of SA-β-gal active cells (*P*<0.05, Figure 3A-3D). All these data indicated that calcitriol has also the activity of inducing cell senescence.

### Calcitriol increases expressions of JMJD3 and p16INK4A

**Figure 4:**
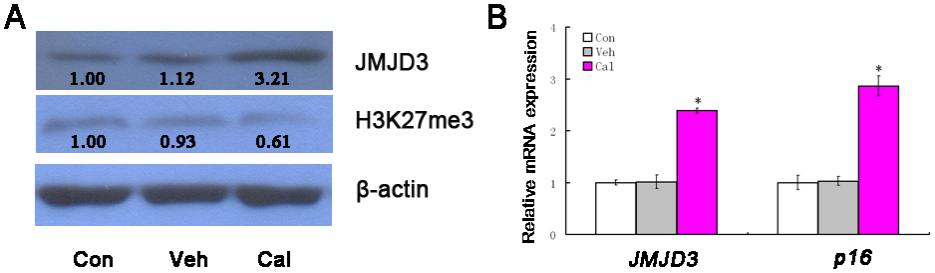
Calcitriol increases the expressions of JMJD3 and p16INK4A 786-O cells were treated with vehicle (Veh) or 100nM calcitriol (Cal) for 48h, and untreated group as control (Con). The expressions of JMJD3 and p16INK4A were determined with qRT-PCR or western blotting. **(A)** The results of qRT-PCR. **(B)** The results of western blotting. *, *P* < 0.05.

We next examined the effects of calcitriol on specific gene expressions. The results showed calcitriol can increase the mRNA and protein content of JMJD3, and at the same time reduce the level of H3K27me3 (Figure 4A and 4B). On the other hand, calcitriol also increased the gene expression of *p16INK4A* (*P*<0.05, Figure 4B). Thus, these results illustrated that calcitriol is important on histone modification and cell senescence in kidney cancer.

### Knockdown of JMJD3 inhibits calcitriol-induced p16INK4A upregulation

**Figure 5:**
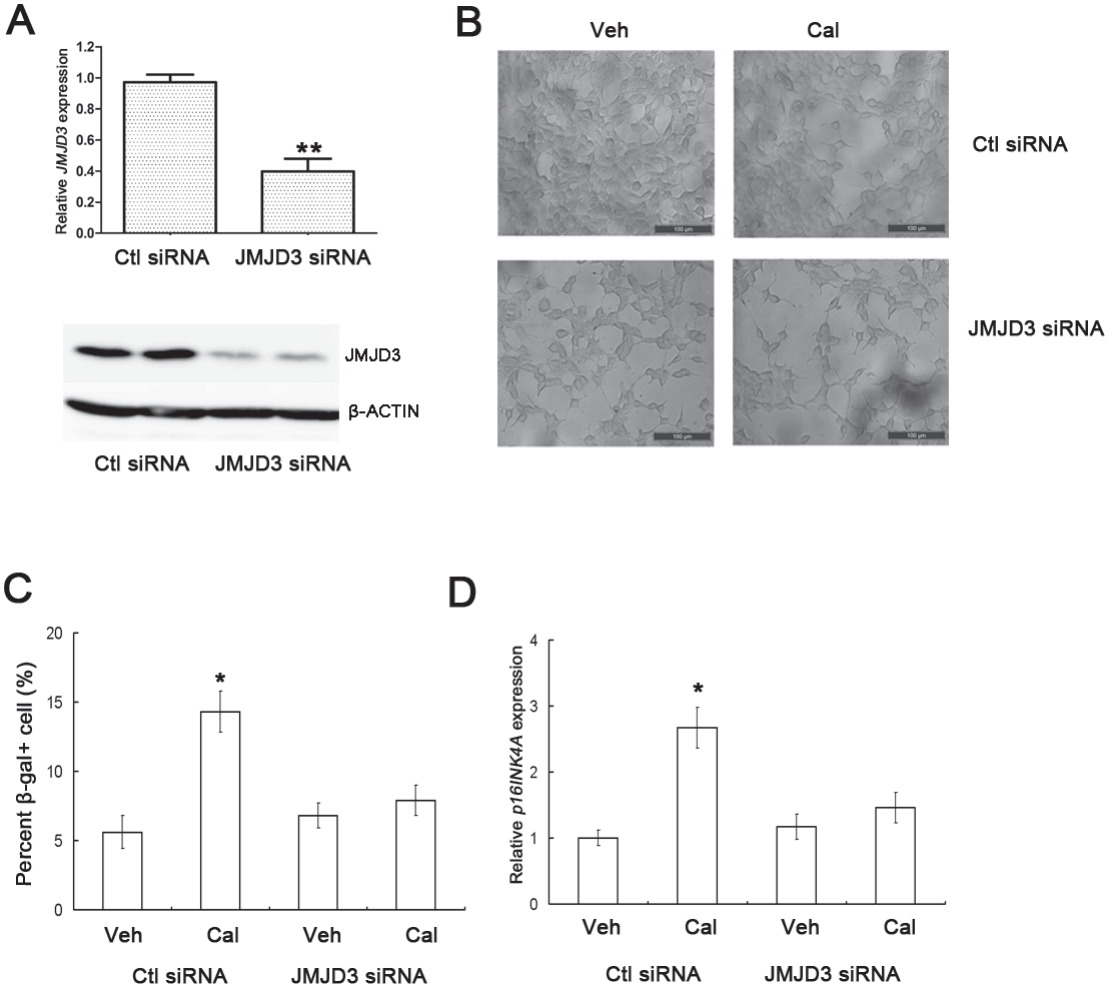
Knockdown of JMJD3 inhibits calcitriol-induced p16INK4A upregulation and cell senescence (**A)** 786-O cells were transfected with JMJD siRNA or control (Ctl) siRNA, and the knockdown effects were determined with qRT-PCR (up) and western blotting (down). (**B)** 786-O cells transfected with Ctl siRNA or JMJD3 siRNA were treated with vehicle (Veh) or 100nM calcitriol (Cal) for 48h, and cell senescence was determined by SA-β-gal staining. (**C)** Quantitative results of cell senescence. (**D)** The expression of p16INK4A in 786-O cells first transfected with Ctl siRNA or JMJD3 siRNA, and then treated with Veh or 100nM Cal for 48h. Scale bar, 100μm. Magnification, 200×. *, *P* < 0.05.

To prove the causal relationship between JMJD3 and p16INK4A, we performed the JMJD3 knockdown experiment. The results showed that knockdown of JMJD3 can inhibit the elevation of *p16INK4A* expression after calcitriol treatment (Figure 5A and 5D). The result also indicated that the induced effect of calcitriol on cell senescence of 786-O was obviously inhibited after JMJD3 knockdown (Figure 5B and 5C). So, these data supported that the biological effect of calcitriol induced cell senescence is partly achieved by JMJD3 mediated *p16INK4A* upregulation.

## DISCUSSION

Calcitriol has a broad variety of anticancer actions, which regulates the cell cycle, induces apoptosis and promotes cell differentiation [22]. In addition, calcitriol can also induce cell senescence of prostate cancer cells [23]. In the study, our data confirm this set of conclusions and thus provide important support for therapeutic use of calcitriol [24]. However, there are still many problems in the clinical application of calcitriol for cancer treatment [25], so further researches on the mechanism of calcitriol are of great significance.

The anticancer effects of calcitriol are mainly mediated via binding to the vitamin D receptor (VDR) which can recruit complexes of either coactivators or corepressors to modulate the transcription of specific genes encoding proteins [26]. A number of histone demethylases are primary targets of VDR and its ligands [27], which participate together in the biological function of calcitriol. JMJD3 is an important H3K27 demethylase and epigenetic regulator, which plays an essential role in many diseases including cancer [28]. Calcitriol can upregulate the expression of JMJD3 [16, 17]. JMJD3 is also a coactivator which can increases gene transcription by catalyzing H3K27me3 demethylation, promoting transcriptional elongation and co-localizing with elongating RNA polymerase II(RNAPII) [29, 30]. Our results indicated that calcitriol up-regulates the expression of JMJD3 in kidney cancer cell, and then JMJD3 further increases transcription of *p16INK4A*. It has shown that vitamin D metabolism is closely related to epigenetic modification such as histone acetylation [31], and the study further expands the view that vitamin D (calcitriol) also regulated histone methylation.

It is widely believed that cell senescence is a tumor suppressor mechanism, which limits tumor progression and determines the outcome of conventional anticancer therapies [32]. Previous studies have demonstrated that JMJD3 can activate *p16INK4A* expression, and then cause p16INK4A-dependent senescence [33, 34]. Our results indicated that calcitriol increases the expression of *p16INK4A* and cell senescence, and the effect can be reduced after JMJD3 knockdown. This fact reflects that JMJD3-mediated upregulation of *p16INK4A* is a cause of calcitriol-induced cell senescence. Cell senescence is also considered to be a potentially important for cancer development, cancer suppression and the response to therapy [35], so it is possible to design new therapeutic approaches to senescence can improve the efficacy and decrease the side effects of cancer therapy [36]. Therefore, our findings provide a new strategy for the treatment of kidney cancer.

**Figure 6:**
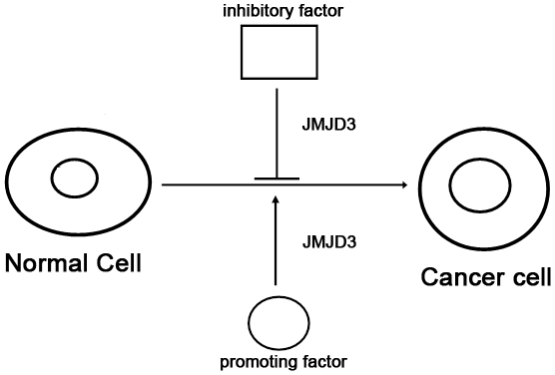
The proposed model of JMJD3 action JMJD3 promotes cancer development by participating in promoting factor signaling pathways. JMJD3 also can inhibit tumor formation by participating in inhibitory factor signaling pathways. The transformation from normal cells to cancer cells can be realized when the activity of the promoting factor is over the inhibitory factor.

There is still some debate about the carcinogenic and antitumor effects of JMJD3, because JMJD3 is overexpressed in some tumors but is lower in other tumors [37, 38]. In mechanism, JMJD3 not only functions as tumor-suppressor by inducing cell senescence [33, 34], but also promotes carcinogenesis via participating in hypoxia signaling pathway [39]. We propose a hypothesis for the converse result (Figure 6). JMJD3 is only a transcriptional activator for gene expression and its role should be determined by the target genes. The regulation of target genes is eventually influenced by external signals. When there are cancer promoting factors, JMJD3 plays a carcinogenic role. Conversely, JMJD3 exerts antitumor activity when inhibitory factors are present. Normal cell can be transformed into cancer cell only when the promoting factors are greater than the inhibitory factors. Based on this hypothesis, it can be concluded that enhancing activity of inhibitory factors such as cell senescence can be regarded as an important strategy for cancer prevention and treatment of early cancer. Because senescence can play a role in tumor suppression in the course of the transformation of a normal cell to a cancer cell [40], JMJD3 mediated senescence induced by calcitriol is an inhibitory factor in tumor development.

Although the epidemiological, preclinical and clinical studies shown that calcitriol has important potential as preventative and therapeutic anticancer agents [41], there are still many problems to be solved for the use of calcitriol in kidney cancer. Many studies have shown that there is abnormal vitamin D metabolism in kidney cancer development and progression [42, 43], which means that vitamin D regulation is beneficial to the treatment of kidney cancer. However, VDR expression is frequently absent or undetectable in cancer tissues of kidney cancer compared to normal tissues [44, 45], which greatly limits the anti-cancer effect of calcitriol. Based on relevant views and results [46], we hypothesize that vitamin D or its analogues are limited in cancer treatment and they should be more used in cancer prevention or in combination with other compounds for cancer treatment. Therefore, the molecular mechanisms and clinical trials of calcitriol need to be further studied before it can be applied to kidney cancer in the future.

In summary, our results demonstrate that calcitriol can promote cell senescence of kidney cancer cells through increasing the expression of JMJD3 and JMJD3-mediated *p16INK4A* upregulation. These findings expand the understanding on the mechanism of JMJD3 action and provide a new strategy for the prevention and treatment of kidney cancer with calcitriol.

## MATERIALS AND METHODS

### Patients and tissue specimens

All cancer and adjacent tissue samples from 36 kidney cancer patients were obtained immediately after surgery. The tumor stage of most of patients was T1 and T2, and there was no obvious metastasis. All patients gave written informed consent. The protocol was approved by the Human Research Ethics Committee of the Fourth Hospital of Hebei Medical University.

### Cell culture and calcitriol treatment

The human embryonic kidney cell HEK293, kidney cancer cells 786-O and ACHN were purchased from cell resource center of Shanghai Institutes for Biological Sciences, Chinese Academy of Science. These cells were maintained in Dulbecco’s modified Eagle’s medium (GIBCO, Grand Island, USA) supplemented with 10% fetal bovine serum (FBS, Hyclone, Logan, USA), 50 U/mL penicillin and 50 μg/mL streptomycin. Cells were cultured in a humidified atmosphere with 5% CO_2_ at 37°C. For calcitriol (Haoyuan chemexpress co., Ltd, Shanghai, China) treatment, calcitriol was first dissolved in 100% ethanol (vehicle), and then added into cell culture medium with given concentration (100 nM).

### JMJD3 knockdown assay

786-O cells were seeded at 70-80% confluency before transfection. Human JMJD3 siRNA and control siRNA were purchased from Genepharma (Shanghai, China), dissolved in sterile DNase/RNase-free water and transfected into 786-O cells using Lipofectamine 2000 (Invitrogen, Carlsbad, CA, USA) at a final concentration of 20 nM/well. The sequences were as follows: control siRNA: 5’-UUCUCCGAACGUGUCACGU-3’; JMJD3 siRNA: 5’- GAGACCTCGTGTGGATTAA-3’.

### Quantitative real-time polymerase chain reaction (qRT-PCR)

Total RNA was extracted from tissues or cells using the Trizol reagent (Invitrogen, China) in accordance with manufacturer’s protocol. The total RNA concentration was determined with Nanodrop 2000 (Thermo Scientific, Wilmington, USA). 1 μg RNA was reversely transcribed into first-strand cDNA using a reverse transcription system (Invitrogen) according to protocol. Then, qRT-PCR was performed in 20 μl reaction mixture containing 10μl of SYBR Premix, 0.5μM of forward and reverse primers, and 1μl template cDNA on LightCycler480 System (Roche, Foster City, CA, USA). The primers of human JMJD3, p16INK4A and β-ACTIN were synthesized by Sangon Biotech (Shanghai, China) and sequences were as follows. JMJD3 primers forward: 5’-CACCCCAGCAAACCATATTATGC-3’; reverse: 3’- CACACAGCCATGCAGGGATT-5’. p16INK4A primers forward: 5’-GAAGGTCCCTCAGACATCCCC-3’; reverse 5’-CCCTGTAGGACCTTCGGTGAC-3’. β-Actin primers forward: 5’-CCACTGGCATCGTGATGGACTCC-3’; reverse: 5’-GCCGTGGTGGTGAAGCTGTAGC-3’. Relative mRNA level of *JMJD3* or *p16INK4A* was normalized to the internal reference *β-Actin*.

### Western blots

The tissues and cells were added radioimmuno-precipitation assay(RIPA) buffer containing the protease inhibitors cocktail (1 mmol/L) and phenylmethylsulfonyl fluoride (100μg/mL), and sonicated to prepare homogenates. Homogenates were centrifuged and supernatants were collected. 50 μg protein was separated by 10% sodiumdodecyl sulfate-polyacrylamide gel electrophoresis (SDS-PAGE) and transferred to polyvinylidene difluoride (PVDF) membranes. The membranes were saturated with 5% skim milk in TBST for 2h and then incubated with primary antibodies at 4°C overnight. The primary antibodies used in this study included rabbit polyclonal antibodies to JMJD3 (1:1500, Abcam, Hong Kong, China), H3K27me3 (1:1,500, Epigentek, Brooklyn, USA) and β-Actin (1:2,500, Sigma, St Louis, USA). The membranes were incubated with HRP-conjugated goat anti-rabbit antibody (1:5,000, Sigma) for 1 h at room temperature and then exposed to enhanced chemiluminescence substrate (Millipore, Rockford, USA), and detection was performed using a film.

### Cell proliferation assay

The cell proliferation was carried out using 3-(4,5-dimethylthiazol-2-yl)-2,5- diphenyltetrazolium bromide (MTT) assay. Briefly, HEK293 or 786-O cells were seed in a 96-well plate for 24 h, and then treated with vehicle or calcitriol. At 0, 24, 48, and 72 h after treatment, cells were added 5 mg/ml MTT and then cultured for another 4 h at room temperature, and lysed in dimethyl sulfoxide (DMSO) for 10 min. The absorbance was measured at a wavelength of 490 nm with an ELISA microplate reader (Bio-Rad, Hercules, CA, USA).

### Cell apoptosis assay

Cell apoptosis was performed with flow cytometry analysis using an Alexa Fluor^®^488 Annexin V/Dead Cell Apoptosis Kit (Invitrogen, Carlsbad, CA, USA) according to the protocols. Briefly, 786-O and ACHN cells were seeded in 6-well plates (1 × 10^5^/well) and treated with vehicle or calcitriol for 48h. The cells were harvested and washed with PBS twice. The cells then were re-suspended in 100 μl 1 × annexin-binding buffer and added 5 μl Alexa Fluor^®^ 488 annexin V plus 1 μl PI working solution in each tube. The tubes were incubated for 15 min at room temperature. Cell apoptosis was analyzed by the flow cytometry (EPICS, XL-4, Beckman, CA, USA).

### Cellular senescence assay

The assay was completed with Cellular Senescence Assay Kit (KAA002, Millipore, USA). The 786-O and ACHN cells were seeded in 6-well plates (1 × 10^5^/well) and treated with vehicle or calcitriol for 48h. The cells were washed with PBS once and added Fixing Solution, and then incubated at room temperature for 15 min. After removing the Fixing Solution and washing with PBS, the cells were added SA-β-gal Detection Solution and incubated at 37°C for 6h. The cells were washed twice with PBS. The blue stained cells were observed and counted under light microscopy.

### Statistical analysis

All experiments were repeated at least three times. The data represent means ± SD from three independent experiments. The differences between two groups including clinical data and cell experiments were analyzed with unpaired two-tailed Student *t* test. *P*<0.05 was considered statistically significant.

## SUPPLEMENTARY MATERIALS FIGURE



## Abbreviations

1,25(OH)_2_VD_3_: 1,25-dihydroxyvitamin D3
JMJD3: Jumonji domain-containing protein 3
VDR: vitamin D receptor
