# Supplementary material for: Calcitriol induces cell senescence of kidney cancer through JMJD3 mediated histone demethylation
Source: Oncotarget. 2017 Oct 26;8(59):100187–95. doi: 10.18632/oncotarget.22124 (PMC5725012; doi:10.18632/oncotarget.22124)
Supplement: Supplementary file 1 [file oncotarget-08-100187-s001.pdf]

Calcitriol induces cell senescence of kidney cancer through JMJD3 mediated histone demethylation

SUPPLEMENTARY MATERIALS

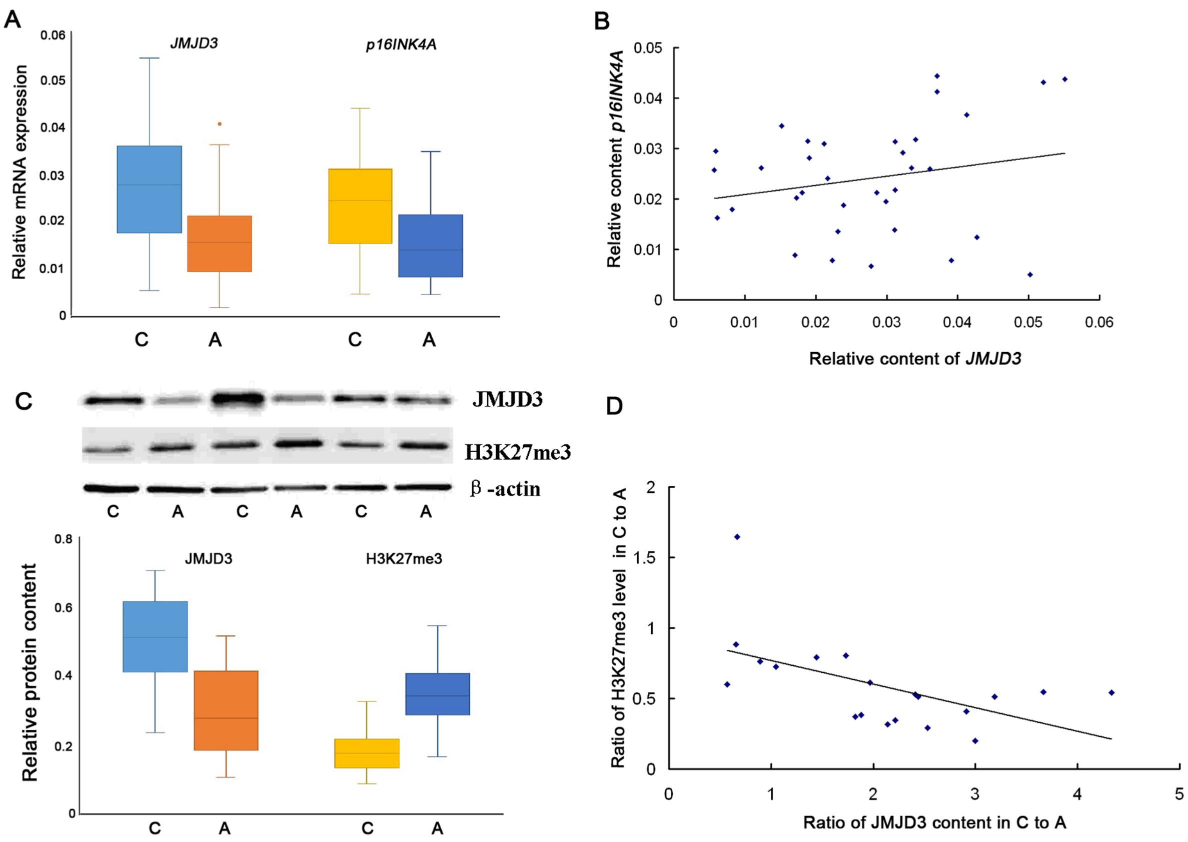

Supplementary Figure 1: The expressions of *JMJD3* and *p16INK4A* in kidney cancer tissues and adjacent tissues. (A) The expressions of *JMJD3* and *p16INK4A* were higher in cancer tissues **(C)** than adjacent tissues (A) from kidney cancer patients (n = 36, $P < 0.05$). **(B)** A positive correlation between *JMJD3* and *p16INK4A* expression in kidney cancer patients. (C) The protein content of JMJD3 was higher in cancer tissues than adjacent tissues, and H3K27me3 level was lower in cancer tissues (n=20, $P < 0.05$). **(D)** A negative correlation between relative levels of JMJD3 and H3K27me3 in cancer tissues to adjacent tissues.
